# ESTPiper – a web-based analysis pipeline for expressed sequence tags

**DOI:** 10.1186/1471-2164-10-174

**Published:** 2009-04-21

**Authors:** Zuojian Tang, Jeong-Hyeon Choi, Chris Hemmerich, Ankita Sarangi, John K Colbourne, Qunfeng Dong

**Affiliations:** 1 The Center for Genomics and Bioinformatics, Indiana University, Bloomington, Indiana, USA

## Abstract

**Background:**

EST sequencing projects are increasing in scale and scope as the genome sequencing technologies migrate from core sequencing centers to individual research laboratories. Effectively, generating EST data is no longer a bottleneck for investigators. However, processing large amounts of EST data remains a non-trivial challenge for many. Web-based EST analysis tools are proving to be the most convenient option for biologists when performing their analysis, so these tools must continuously improve on their utility to keep in step with the growing needs of research communities. We have developed a web-based EST analysis pipeline called ESTPiper, which streamlines typical large-scale EST analysis components.

**Results:**

The intuitive web interface guides users through each step of base calling, data cleaning, assembly, genome alignment, annotation, analysis of gene ontology (GO), and microarray oligonucleotide probe design. Each step is modularized. Therefore, a user can execute them separately or together in batch mode. In addition, the user has control over the parameters used by the underlying programs. Extensive documentation of ESTPiper's functionality is embedded throughout the web site to facilitate understanding of the required input and interpretation of the computational results. The user can also download intermediate results and port files to separate programs for further analysis. In addition, our server provides a time-stamped description of the run history for reproducibility. The pipeline can also be installed locally, allowing researchers to modify ESTPiper to suit their own needs.

**Conclusion:**

ESTPiper streamlines the typical process of EST analysis. The pipeline was initially designed in part to support the *Daphnia pulex *cDNA sequencing project. A web server hosting ESTPiper is provided at  to now support projects of all size. The software is also freely available from the authors for local installations.

## Background

Expressed sequence tags (ESTs) are generated by single-pass sequencing of complementary DNA (cDNA) [1]. Because ESTs correspond to the transcribed regions of a genome, EST sequencing has been a common strategy for gene discovery – especially for organisms with complex genomes. For example, many agriculturally important plants (*e.g*., sunflower, wheat) have enormous genomes containing many repetitive elements and large intergenic regions. For these taxa, EST sequencing remains (for now) the only efficient way to discover genes on genome-wide scale, since the repetitive elements still pose an unsolved challenge for whole genome assembly. Even for species with draft genome sequences, ESTs remain the gold standard for accurate gene structure annotations (delineating intron-exon and gene boundaries) and serve a variety of biological research applications (reviewed in [2-4]). Because of improved reliable protocols for cDNA library construction, normalization and sequencing, generating EST data is now standard practice. As a result, the number of EST sequences is growing at an ever-increasing pace for diverse organisms [5]. After EST sequencing, the next step is to analyze the generated EST data. Typical EST analysis involves (i) base calling to convert raw chromatograms generated by DNA sequencers into human-readable sequences, (ii) data cleaning that removes cloning vector, adaptor and bacterial host sequence contamination, (iii) assembling individual EST sequences into contigs that reduce redundancy and represent a unique gene set, (iv) functional annotations of the potential encoded proteins by sequence similarity to annotated proteomes, and (v) designing microarray oligonucleotide probes from the EST sequences for expression profiling. For many of the above tasks, bioinformatics groups that specialize in EST data (*e.g*., PlantGDB [6]) usually implement their own in-house EST analysis pipelines. However, those pipelines are generally not portable or accessible to outside users, due to specialized hardware requirement (such as parallel computers). Many biologists with smaller-scale EST projects can not afford dedicated bioinformatics teams or computational clusters. Therefore, individual biologists can face significant challenges to process and analyze their EST data. These include identifying, installing and executing the proper computer programs for each step. This is especially challenging if data processing requires additional programming (*e.g*., converting the output of one program to the required format for input into the next program). In most cases, significant computational resources are also needed (*e.g*., fast computers with enough memory and disk space). Therefore, web-based EST analysis pipelines are critical for biologists to perform their analysis simply, via a web browser, without unnecessary technical hassles. Although some web-based tools for EST analysis are becoming available, their scope and capacities need continuous improvements. To enrich the biologists' toolkit, we have developed a web-based computational pipeline called ESTPiper, which streamlines typical EST analysis steps. In the sections below, we discuss the technical implementation of ESTPiper, its unique features compared to other web-based EST analysis tools, and the application of ESTPiper in support of the *Daphnia pulex *genome sequencing project .

## Implementation

**Figure 1:**
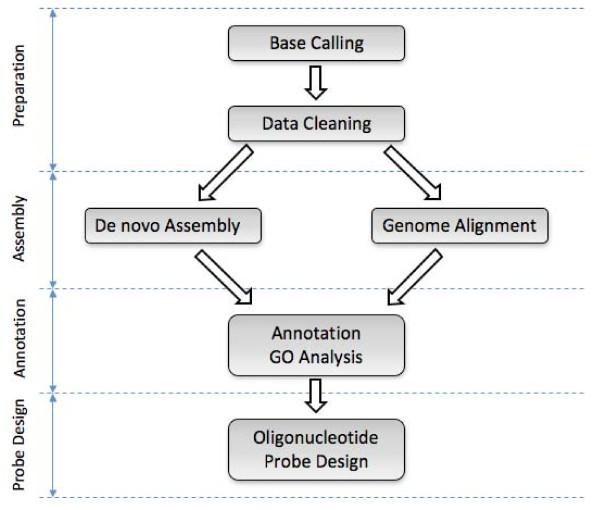
**Schematic overview of ESTPiper**. See text for detailed description.

The ESTPiper flow chart is illustrated in figure 1. The intuitive web interface guides the researcher through each step: base-calling, data cleaning, assembly, genome alignment, annotation, GO functional analysis, and microarray oligonucleotide probe design. At each step, the user sets the parameters to be used by the underlying analysis programs. Once the computation is completed, the user is notified via email and given a URL for viewing and downloading the results. For convenience, the results are temporarily stored on our server for 60 days. Each specific component of ESTPiper is described below.

### Base calling

ESTPiper incorporates the Phred program [7] for base calling with quality scores, which is the *de facto *standard program for converting DNA sequence trace files (*i.e*., chromatograms) generated by the Sanger method into nucleotide sequences. To speed up the file upload for large numbers of trace files, ESTPiper only accepts compressed data files in either .*ab1 *or .*scf *format and produces the sequence and the corresponding quality file in FASTA format.

### Data cleaning

To obtain high quality EST assemblies, contaminant sequences are properly identified and removed. These include sequences from ligated adaptors, cloning vectors and the bacterial host. ESTPiper first invokes the commonly used LUCY program [8] for vector removal and to trim low quality regions at both ends of sequence reads. In addition, the user has the option to trim polyA/T tails from the sequence reads, which is a necessary step to avoid mis-assembly but optional if the user intends to identify transcriptional termination sites on the genome. Finally, the sequences are compared against bacterial genomes (downloaded from GenBank FTP site [9]) and other adapters and primers (UniVec [10]) based on stringent BLAST searches (*i.e*., E-value cutoff 1 × 10^-20^). The output of this step is the cleaned sequence and quality files in FASTA format.

### Assembly

ESTs usually correspond to only partial cDNA sequences and they are typically redundant, even when normalized during library construction. Therefore, overlapping EST sequences are commonly assembled to derive a set of unique putative genes (unigenes). ESTPiper provides *de novo *assembly using the popular CAP3 program [11] to assemble ESTs into contigs based on mutual percent identity over a minimum number of overlapping bases. In addition, if the 5' and 3' ESTs derived from the same cDNA clones follow the standard naming convention (*e.g*., .fwd and .rev for sequences generated from forward and reverse sequencing primers, respectively), such clone-pair information can be used to produce unigene clusters that include non-overlapping contigs. For this purpose, ESTPiper performs single-linkage clustering by default. The user may choose a more stringent clustering criterion, *i.e*., at least two EST clones must be shared for linking two contigs together in order to reduce potential false linkages (similar to other practice, *e.g*., [12]).

### Genome alignment

If a draft genome sequence for the species of interest is available, ESTs are routinely aligned to genomic DNA for gene discovery, for annotation of intron-exon structures, and for identifying alternative splice forms. For such purposes, we have also implemented a genome-alignment module. Specifically, the BLAT program [13], which is designed to align native or closely related ESTs to the genome, is called by ESTPiper to perform spliced alignment of each EST against user-supplied genome sequences. If an EST sequence matches multiple genomic loci, only the best match is considered as the cognate match. After ESTs are aligned to genomic DNA, the mapping coordinates (*i.e*., the start and end position of each aligned EST on genomic scaffolds) allow the user to cluster overlapping ESTs into unigene sets. Genome-based EST clustering is usually considered more accurate than *de novo *assembly [12,14-16]. Without draft genome sequences for guidance, *de novo *assemblers solely consider the pairwise EST sequence overlap, and may mistakenly assemble different transcripts from paralogous genes into the same cluster, which is a serious problem for species that have extensive gene duplicates. Moreover, *de novo *assemblers often disconnect alternative transcripts derived from the same gene locus into different clusters, thus overestimating the number of expressed genes. Therefore, we have implemented a genome-guided strategy for clustering the mapped ESTs similar to other published studies (*e.g*., [12,16]). Particularly, ESTs aligned to the same genomic locus are clustered based on user-supplied parameters, *e.g*., minimum number of overlapping nucleotides between the neighboring aligned EST sequences. Then, transcript sequences can be derived based on the exons defined by each matched EST produced by the BLAT program. Specifically, ESTPiper invokes the well-known PASA package to generate contigs for each alternative splice variant by merging sets of compatible overlapping EST alignments [17]. If multiple splice variants are identified, all will be reported. However, only the longest splice variant from each gene cluster is selected as the representative of the gene transcript for the following modules (*i.e*., functional annotation and microarray oligonucleotide probe design).

### Functional Annotation

The next natural step when processing ESTs is to identify the potential protein products encoded by the clustered unigene sequences, particularly whether they are similar to known sequences in proteomic databases. Therefore, each assembled contig sequence is searched against the UniProt protein database [18] using the BLAST program [19] in ESTPiper. A local copy of the UniProt database is automatically synchronized with the UniProt server via a monthly cron job. The UniProt database has the advantage over other comprehensive datasets for annotation of providing gene ontology (GO) terms in its protein records [20]. GO terms associated with the top statistically significant database sequence matches are propagated in ESTPiper to the contig sequence following common practice (*e.g*., [21]). The GO terms allow biologists to conveniently summarize the gene product attributes of their sequences with controlled vocabulary. ESTPiper allows the user to create a summary of GO terms in tabular format based on different GO categories (*i.e*., biological process, molecular function, and cellular component) and at a user-selected GO hierarchical level. ESTPiper also allows the user to input a list of GO terms (*e.g*., GO terms derived from genes unique to a particular EST library) and reference set (*e.g*., GO terms corresponding to the entire transcriptome), and to perform statistical analyses that identify overrepresented functional attribute terms. Specifically, the p-value of each GO term is calculated based on the hypergeometric test with Benjamini and Hochberg multiple testing correction [22].

### Microarray oligonucleotide probe design

Despite increasing popularity of whole-genome tiling arrays and expression profiling by direct sequencing, oligonucleotide arrays remain an efficient and cost effective tool for studying co-transcriptional biases of genes on a large scale for species without full-genome sequence information. Therefore, designing microarray oligonucleotide probes is a critical task for biologists to create gene chips for their functional genomic investigations. Previously, our Center conducted an extensive survey to compare the existing microarray probe design programs [23] and chose the OligoPicker program [24] to successfully design microarray probes within ESTPiper for a number of expression profiling studies in different species (*e.g*., *Coprinus cinereus, Daphnia pulex*). The coding strand (*i.e*., sense strand) of each assembled sequence must be pre-defined before designing microarray probes, which is seldom straightforward. In ESTPiper, the coding strand is determined by a simple two-tier strategy. First, sequences on DNA strands that match to the protein database (*e.g*., through the above *Functional Annotation *module), are confidently determined from the BLAST output. If a sequence has no match to protein sequences (*e.g*., EST sequences representing novel species-specific genes), the sequence is passed to the OrfPredictor program [25], which identifies the longest open reading frame to predict the coding strand.

### Automated Pipeline

Each of the above steps is completely modularized. Therefore, depending on the user's specific needs, multiple entry points into the pipeline are possible. For example, instead of starting with base calling for processing trace files, the user can simply upload pre-processed FASTA-format sequence files and quality scores (perhaps generated by others) into our pipeline for assembly. Similarly, intermediate results can be downloaded from our pipeline (*e.g*., assembled contig sequences) to be imported within other preferred computer programs for further analysis (*e.g*., functional annotation). In addition, ESTPiper provides an option for the user to select and combine multiple (or all) modules automatically. Data can be effectively transferred among adjacent modules without any human intervention. Parameters can be saved for repeated use, *i.e*., the user can input previously returned parameter files for multiple new runs with different source data.

## Results and discussion

### Comparison with other existing EST analysis programs

A number of standalone software packages are available for EST data analysis [26-37]. However, those programs require researchers to install and maintain the software locally, which many biologists find inconvenient (*e.g*., certain prerequisites can present a serious challenge to install and update, even for bioinformaticians). Instead, biologists often prefer web-based analysis tools, where data can be uploaded on a host machine and the analysis can be performed through an easy-to-use interface. Therefore, several online tools have been published recently that simplify computational tasks. Although helpful, none of the existing web-based tools offer a comprehensive EST analysis workflow. For example, many tools are limited in scope: *e.g*., OREST [38] is only designed for processing mammalian and fungal sequences, not necessarily applicable to other research communities. In addition, OREST does not provide some of the critical modules in ESTPiper (*e.g*., *de novo *EST assembly, microarray probe design). For general-purpose EST analysis tools, preAssemble [39] and WebTraceMiner [40] specialize in base calling and quality trimming but do not provide assembly or annotation functionality. EGassembler [41] mainly focuses on *de novo *EST assembly but users must perform base calling and annotation elsewhere. ESTExplorer [42] and ESTpass [43] extend EGassember with additional functional annotations, but these programs lack genome alignment and microarray probe design functionality. Here, we present an alternative EST analysis pipeline, ESTPiper, for research communities. In addition to streamlining the steps of base calling, quality trimming and removing contaminant sequences, assembly, annotation and GO analysis, ESTpiper provides two unique modules compared to existing web-based EST analysis tools: (i) genome alignment and (ii) microarray probe design. As discussed above, both functions are now standard practice in a typical EST project. However, these functions are not available in existing EST analysis tools (Table 1). Although standalone web servers exist for similar tasks (*e.g*., e2g [44] for genome alignment and PROBEmer [45] for probe design), ESTPiper integrates such functionalities into a comprehensive pipeline with additional enhancements. For the genome alignment module, the existing tools for EST to genome alignment do not provide customizable clustering function. ESTPiper allows users to specify how ESTs aligned to the genome should be clustered, by defining a minimum distance between the neighboring aligned EST sequences. Moreover, such clustering can be improved by providing clone pair information. For the probe design component, all existing probe design software requires researchers to identify the coding strand before executing. However, for a large number of EST-derived sequences, it is not trivial for biologists to determine the correct strand for each. Therefore, we integrated the database similarity search (*i.e*., BLAST) and *ab initio *prediction (*i.e*., OrfPredictor) for coding strand determination. In addition, we also enhanced the *de novo *assembly function. Specifically, beyond simply invoking CAP3 as other services do, ESTpiper provides users an option to perform single-linkage clustering based on clone-pair constraints. This allows the user to better define a true set of unigenes (*i.e*., ESTs derived from the same cDNA clones, even if the sequences do not overlap). Furthermore, some of existing web-based tools allow researchers to process only relatively small input files. For example, ESTpass imposes an upper limit of 10,000 ESTs (or 20 Mbyte file size) on files uploaded to their web server. We impose no file size limit for ESTPiper. However, we do recommend that users input less than 100,000 EST sequences to ensure successful *de novo *assembly without running out of computer memory on our current server. Yet we have nonetheless successfully assembled more than 150,000 *Daphnia *ESTs with our present configuration.

**Table 1:** Comparison of the available features of ESTPiper with other web-based EST analysis tools.

**Web-based EST analysis pipeline**	**preAssemble**	**EGassembler**	**ESTExplorer**	**ESTpass**	**WebTraceMiner**	**ESTPiper**
**Base calling**	Yes	No	No	No	Yes	Yes
**Data cleaning**	Yes	Yes	Yes	Yes	Yes	Yes
**De novo assembly**	No	Yes	Yes	Yes	No	Yes
**Genome alignment**	No	No	No	No	No	Yes
**Annotation**	No	No	Yes	Yes	No	Yes
**GO analysis**	No	No	Yes	Yes	No	Yes
**Probe design**	No	No	No	No	No	Yes

### Other features

Our workbench is designed for biologists to perform and document computational analysis on EST data. Computational analyses in ESTPiper are documented for reproducibility. For example, the percent identity cutoff limits used by CAP3 program for assembly are recorded, which determines the resulting contigs. Therefore, ESTPiper provides the user with a complete, time-stamped description of ESTPiper's usage history (*e.g*., the programs, parameters, input data, and corresponding results). We believe that this feature will greatly facilitate tracking results, especially if the user initiates several rounds of trial-and-error analyses, experimenting with different program and parameter combinations in order to obtain the highest quality results. Finally, unlike many other web-based tools that are not portable, the user can download and install ESTPiper on local computers. For example, advanced users may use the core ESTPiper code to process the EST data without having to navigate through the web interface, or they can integrate ESTPiper into their own customized pipeline (*e.g*., replace any individual modules in ESTPiper with their preferred analysis programs).

### Application

We applied ESTPiper to process and analyze a large set of *Daphnia pulex *EST data as part of the *Daphnia *Genomics Consortium sequencing project. We began our data analysis with 151,111 EST sequences that were filtered from an initial set of 219,948 trace files that were generated by sequencing 37 cDNA libraries for discovering condition specific gene transcripts (detailed analysis of these results is presented elsewhere). After data cleaning, ESTPiper returned 151,013 high-quality sequences. PolyA/T tails were further removed; the minimum length of continuous polyA/T was set to 9 bp, the maximum number of mismatches within the polyA/T region was 3, the searching range of polyA/T was limited to 50 bp from both ends of the sequence. We also configured ESTPiper to remove sequences with at least 30 bp continuous A/T or adaptors occurring in the middle of sequence reads to avoid potential chimerical clones. Furthermore, mitochondrial sequences and contaminated *E. coli *sequences were identified and removed based on BLAST similarity search (E-value cutoff 1 × 10^-10^). Finally, resulting sequences less than 100 bp were also removed. A total of 148,410 high-quality ESTs were therefore used in subsequent steps of our analysis.

We conducted both a *de novo *assembly using the CAP3 program and an assembly based on alignment to the Dappu v1.1 draft genome sequence assembly (September, 2006). First, by feeding the cleaned ESTs into the *de novo *CAP3 assembly program (with the parameters -*p 95 -o 49 -t 10000*), 23,470 contigs and 14,014 singletons were generated, and 26,265 unigene clusters were derived based on clone-pair constraint. Second, for genome-based assembly, ESTs were first aligned to the *Daphnia *genome using the BLAT program (with the parameter *minIdentity *= *95*). If an EST sequence matched multiple genomic loci, only the best match was considered as the cognitive match. Out of 148,410 ESTs, 113,931 ESTs matched to the genome sequence. ESTs were clustered based on their overlapping matching positions on the genome. We required that two neighboring ESTs be considered part of the same cluster if they shared at least 40 bp minimum overlap. A total of 14,891 unigene sets were derived. For genes identified from each EST library, ESTPiper matched them to UniProt using BLASTX (E-value cutoff is 1 × 10^-20^). The GO term associated with the top matches to the protein database were also created for different libraries. Statistic analysis of GO terms overrepresented in each library was performed using the entire EST collection as a reference. Finally, a 10,000 element *Daphnia *cDNA microarray (Generation-3) was produced with the oligonucleotide probes designed based on ESTPiper. The microarray has been successfully applied by the *Daphnia *research community to study *Daphnia *gene expression under different environmental stress conditions (data will be published elsewhere).

## Conclusion

Web-based tools are most convenient for biologists to effectively process large EST data sets. To supplement the existing tools, we have developed a comprehensive web-based EST analysis pipeline called ESTPiper that streamlines the numerous EST analysis components and offers unique features such as genome alignment and microarray probe design.

## Availability and requirements

The ESTPiper program is freely accessible, using a web browser at . We recommend that users provide their email address when they upload their data. Then, once their submitted jobs are finished, emails will be automatically sent to the users with the instruction for retrieving their results. The software is also available from the web site for local installation. Currently, ESTPiper is installed on a virtual machine hosted on a Sun X4450 with four 2.4 GHz CPUs, each CPU having four cores for 16 total cores. The machine has 32 GB of memory. There are two 10 K RPM SAS system disks in a mirrored ZFS pool, and all project/app storage is done over NFS via dedicated gigabit Ethernet. At our Center, we can easily migrate ESTPiper among our virtual servers as resource requirements change.

**Project name: **ESTPiper

**Project home page: **

**Operating systems: **Local installation requires Linux/UNIX.

**Programming language: **Perl, JavaScript, JAVA

**License: **The software is under the Apache license 2.0.

## Authors' contributions

ZT designed and implemented ESTPiper and its web server. JHC contributed to the system design and provided technical assistance with the software implementation. CH critically enhanced the web interface, on-line descriptions, and the probe design module. AS and CH improved the genome alignment module. QD and JC conceived the project and guided the development process. QD and ZT prepared the manuscript. All authors read and approved the final manuscript.
